# Experimental Investigation of Deformable Gel Particles (DGPs) for Plugging Pan-Connected Interlayer Channels in High-Water-Cut Reservoirs

**DOI:** 10.3390/gels11090686

**Published:** 2025-08-27

**Authors:** Wenjing Zhao, Jing Wang, Tianjiang Wu, Ronald Omara Erik, Zhongyang Qi, Huiqing Liu

**Affiliations:** 1 State Key Laboratory of Petroleum Resources and Prospecting, China University of Petroleum, Beijing 102249, China; zhaowenjing1_cq@petrochina.com.cn (W.Z.); ronalderikomara@yahoo.com (R.O.E.); 2024310198@student.cpu.edu.cn (Z.Q.); liuhq@cup.edu.cn (H.L.); 2 Oil and Gas Technology Research Institute of Changqing Oilfield, China National Petroleum Corporation, Xi’an 710018, China; wutianjiang_cq@petrochina.com.cn

**Keywords:** deformable gel particles, interlayer flow channel, partially developed interlayer, physical simulation

## Abstract

Pan-connected interlayers are widely present in oil reservoirs, forming flow channels at different positions. However, conventional profile control agents struggle to plug deep interlayer channels in reservoirs, limiting the swept volume of injected water. Additionally, a clear methodology for physically simulating pan-connected reservoirs with interlayer channels and calculating interchannel flow rates remains lacking. In this study, a physical model of pan-connected interlayer reservoirs was constructed to carry out deformable gel particles (DGPs) plugging experiments on interlayer channels. A mass conservation-based flow rate calculation method for interlayer channels with iterative solution was proposed, revealing the variation law of interlayer channel flow rates during DGP injection and subsequent water flooding. Finally, oil displacement and DGP profile control experiments in pan-connected interlayer reservoirs were conducted. The study shows that during DGP injection, injected water enters the potential layer through interlayer channels in the middle and front of the water-channeling layer and bypasses back to the water-channeling layer through channels near the production well. With the increase in DGP injection volume, the flow rate of each channel increases. During subsequent water flooding, DGP breakage leads to a rapid decline in its along-path plugging capability, so water bypasses back to the water-channeling layer from the potential layer through all interlayer channels. As the DGP injection volume increases, the flow rate of each channel decreases. Large-volume DGPs can regulate interlayer channeling reservoirs in the high water cut stage. Its effectiveness mechanism involves particle migration increasing the interlayer pressure difference, which drives injected water to sweep from the water-channeling layer to the potential layer through interlayer channels, improving oil recovery by 19.74%. The flow characteristics of interlayer channels during DGP injection play a positive role in oil displacement, so the oil recovery degree in this process is greater than that in the subsequent water flooding stage under each injection volume condition. The core objective of this study is to investigate the plugging mechanism of DGPs in pan-connected interlayer channels of high-water-cut reservoirs, establish a method to quantify interlayer flow rates, and reveal how DGPs regulate flow redistribution to enhance oil recovery.

## 1. Introduction

The interlayer plays a crucial role in determining the distribution and migration of oil and water, as emphasized by recent studies [1,2]. During the combined production process in multi-layer heterogeneous reservoirs, an underdeveloped interlayer can lead to the formation of interlayer channels (ICs). This, in turn, can significantly impact the efficiency of the overall reservoir development. Most studies have investigated interlaminar flow via numerical simulations and theoretical models, but these methods insufficiently capture interlayer flow dynamics [3,4,5]. Theoretical findings are particularly difficult to validate experimentally because recreating and observing interlayer channels under laboratory conditions poses significant challenges. As a result, experimental studies on the behavior of heterogeneous reservoirs in the presence of interlayer flow are relatively scarce.

For instance, Farshad Rezaeiakmal [6] used a visualization model to explore the influence of interlayer flow channels on foam flooding. Zhang [7] applied the Arqi method to compare combined production with layer-by-layer development, revealing that the latter was ineffective in enhancing interlayer flow in deeper reservoir sections. In another study, Lu et al. [8] used a small layer suction metering device to examine the impact of interlayer flow channels on the efficiency of water flooding, considering the variations in liquid volume within the intermediate container. However, this experiment was limited by the inconsistent sensitivity of the piston within the intermediate container, which likely compromised the accuracy of the results. In summary, existing research has not yet provided precise methods for quantitatively characterizing interlayer flow dynamics. Thus, a key challenge for future research lies in developing accurate empirical techniques for analyzing interlayer flow behavior.

The profile control in heterogeneous reservoirs becomes even more challenging during the high water cut stage due to the presence of interlayer channels. These channels not only reduce the effectiveness of profile control but also complicate water shutoff efforts. When a profile control agent is injected, subsequent water flooding can occur. However, the injected water may flow back through the interlayer channels to the dominant seepage paths or water channeling layers, thereby reducing the effectiveness of profile control. To mitigate this issue, it is essential to plug interlayer channels as effectively as possible for optimal reservoir management. But there is limited research focused on profile control strategies for heterogeneous reservoirs with interlayer channels during high water cut conditions. Currently, many studies rely on control agents designed to provide continuous medium profiles [9,10,11]. However, these agents have limitations, particularly in deep migration within the reservoir. Disadvantages include their susceptibility to viscous fingering in subsequent water slugs [12] and their limited effectiveness near the injection well. As a result, water can bypass the control agent and return to the water channeling layer. Consequently, more effective profile control methods are urgently needed for heterogeneous reservoirs with interlayer channels, particularly during the high water cut stage.

Deformable gel particles (DGPs) possess elastic characteristics that enable deep transport within the reservoir. Compared to other profile control agents, DGPs offer distinct advantages for deep profile control, as demonstrated in laboratory experiments [13,14] and field applications [15,16,17,18,19]. In theory, DGPs are better suited for reservoirs with deep interlayer channels compared to other control agents. Elsharafi and Bai [20] proposed that DGPs are particularly effective in heterogeneous reservoirs without interlayers due to their minimal impact on formation damage. Yang et al. [21] suggested using different particle positions to achieve plugging within interlayer channels. However, their evaluation was limited to friction flow as a single parameter, which may not fully account for the flow dynamics in multiple interlayer channels. In contrast, Ji et al. [22] conducted research using acid particles to block gas channeling in reservoirs without interlayers. The study revealed that DGPs effectively enhanced oil recovery in reservoirs with interconnected layers, offering insights into their potential for optimizing oil recovery in similar reservoirs. Interlayer channels vary within a reservoir, making it essential to understand the deep migration of DGPs for effective reservoir regulation [21]. Current research has mainly focused on the compatibility between DGPs and reservoirs [23,24], as well as their working mechanisms [25,26].

In this study, sandpacks were designed as physical models with multiple measuring points to capture various flow channels. By combining this design with flow calculations, we were able to identify the plugging patterns when DGPs were used. The findings of this study offer valuable insights into the impact of different factors on the recovery efficiency of interconnected reservoirs with multiple layers. These results have significant implications for the effective control of injection profiles in heterogeneous reservoirs with interlayer flow channels during the high water cut stage and provide essential guidance for future research in this area.

## 2. Results and Discussion

### 2.1. Interlayer Flow Dynamics During DGP Injection

#### 2.1.1. Influence of Diameter Ratio of DGP to Pore Throat on Interlayer Flow Dynamics During DGP Injection

**Figure 1:**
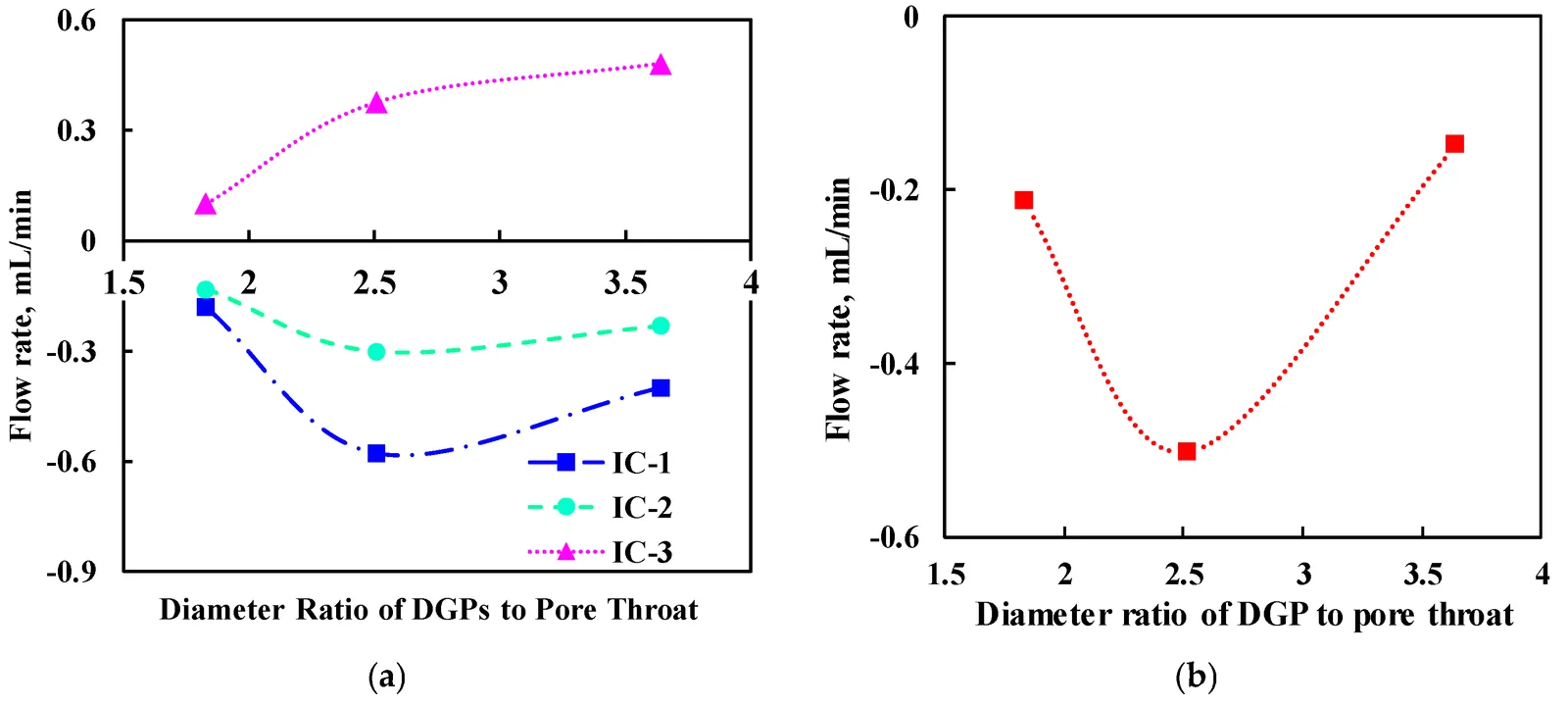
Variation of interlayer channel flow during DGP injection under different diameter ratio of DGP to pore throat. (**a**) flow rate variation of interlayer channels. (**b**) variation of total interlayer flow rate.

Based on the experimental results from Groups 1 to 3 in Table 1, the flow rate variations in the interlayer channels during DGP injection were calculated using the method described earlier, as shown in Figure 1. An analysis of the flow directions in Figure 1a reveals that, regardless of the diameter ratio of DGP to pore throat, the flow direction in the interlayer channel near the production well (interlayer channel-3) consistently moves from the low-permeability layer to the high-permeability layer. This behavior suggests that particle breakage is a significant transport mechanism for DGPs [27]. It can be inferred that the weaker effect of particles near the production well, due to particle breakage and percolation, leads to lower sealing strength in this region, which explains the observed flow characteristics in the interlayer channel near the production well. In contrast, the increased flow resistance in the first half of the high-permeability layer after particle injection results in a higher interlayer pressure difference, driving flow from the high-permeability layer to the low-permeability layer. As shown in Figure 1b, in an interlayer-connected reservoir with a barrier layer, the overall flow direction in the interlayer channels shifts from the high-permeability layer to the low-permeability layer, indicating that flow rates in channels 1 and 2 dominate. The particles have better mobility in the high-permeability layer when the diameter ratio of DGP to pore throat is small, leading to a smaller interlayer pressure difference. Under these conditions, the flow rates in the three interlayer channels are the lowest compared to other diameter ratios. Although small particles possess strong migration capabilities (allowing them to pass through pore throats directly), their weaker action strength results in more particles being released from the high-permeability layer.

Conversely, a larger diameter ratio causes most of particles to be retained at the injection end of the porous media, forming a filter cake. This filter cake can impede subsequent water injection. When the diameter ratio of DGP to pore throat is 2.51, the particles can deform and break, allowing them to penetrate deeper into the high-permeability layer, which increases flow resistance. As a result, the pressure differences across various parts of the porous media become larger and more uniform under these conditions. This leads to the largest interlayer pressure difference and flow rate, with the total interlayer flow rate (in absolute terms) reaching 1.26 mL/min.

#### 2.1.2. Influence of Injection Rate on Interlayer Flow Dynamics During DGP Injection

**Figure 2:**
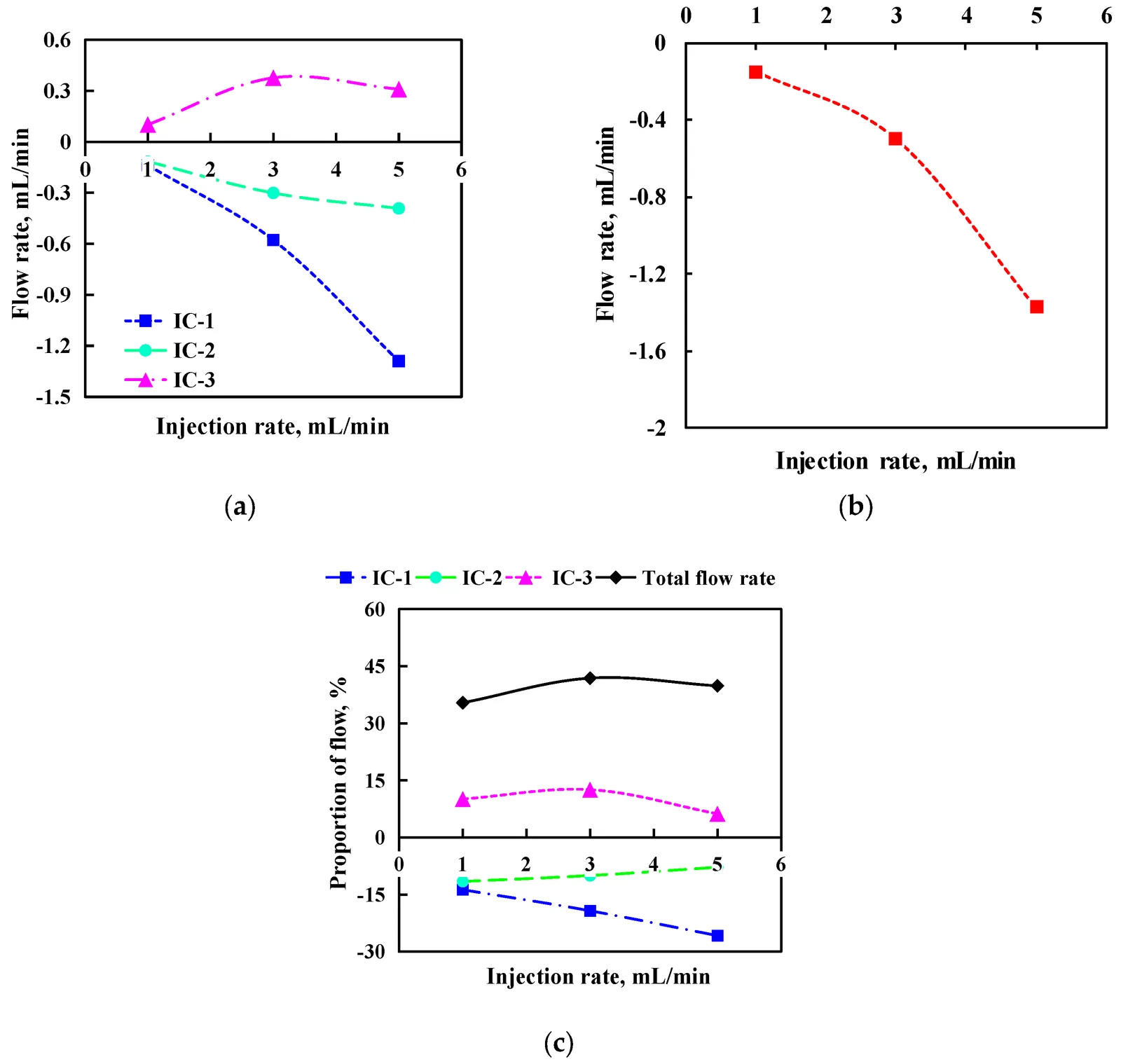
Variation of interlayer channel flow during DGP injection under different injection rate. (**a**) Flow rate variation of interlayer channels; (**b**) variation in total interlayer flow rate. (**c**) Proportion of flow rate of each interlayer channel.

The changes in the interlayer flow rate, calculated from the experimental results of Groups 2, 4, and 5 in Table 1, are presented in Figure 2. Since the injection rate is the primary variable in this part, the absolute flow rate alone does not adequately capture the interlayer flow behavior. Therefore, the interlayer flow rate is normalized by dividing it by the corresponding injection rate, as shown in Figure 2c. Similar to the flow direction observed during DGP injection under varying diameter ratios of DGP to pore throats, the flow pattern under different injection rates reveals that only the interlayer channel near the production well flows from the low-permeability layer to the high-permeability layer. In contrast, the other two channels exhibit flow from the high-permeability layer to the low-permeability layer due to the pressure differences. Building on the analysis of particle plugging, crushing, and migration patterns under different injection rates presented in previous research [27], it can be concluded that when DGPs are injected at a low rate (1 mL/min), shear crushing is less frequent, and particles primarily migrate through deformation within the porous media. This results in a more uniform effect in the high-permeability layer. However, this also leads to smaller interlayer pressure differences and lower interlayer flow rates during the injection process, with the total interlayer flow rate reaching only 0.35 mL/min. This has a minimal impact on the mobilization of remaining oil in the low-permeability layer during particle injection.

Conversely, high-rate DGP injection results in an increase in interlayer pressure differences at the front of the porous media, which leads to higher interlayer flow rates. When the injection rate is 5 mL/min, the total flow rate increases to 1.99 mL/min, accounting for 39.83% of the total injected volume. However, high-rate injection also leads to significant particle crushing, which reduces the effectiveness of particles in deeper reservoir regions. As a result, a large volume of water flows from the high-permeability layer to the low-permeability layer through the first two interlayer channels, causing the flow rate (0.31 mL/min) in the channel near the production well to be lower than in the other two channels.

#### 2.1.3. Influence of Injection Volume on Interlayer Flow Dynamics During DGP Injection

**Figure 3:**
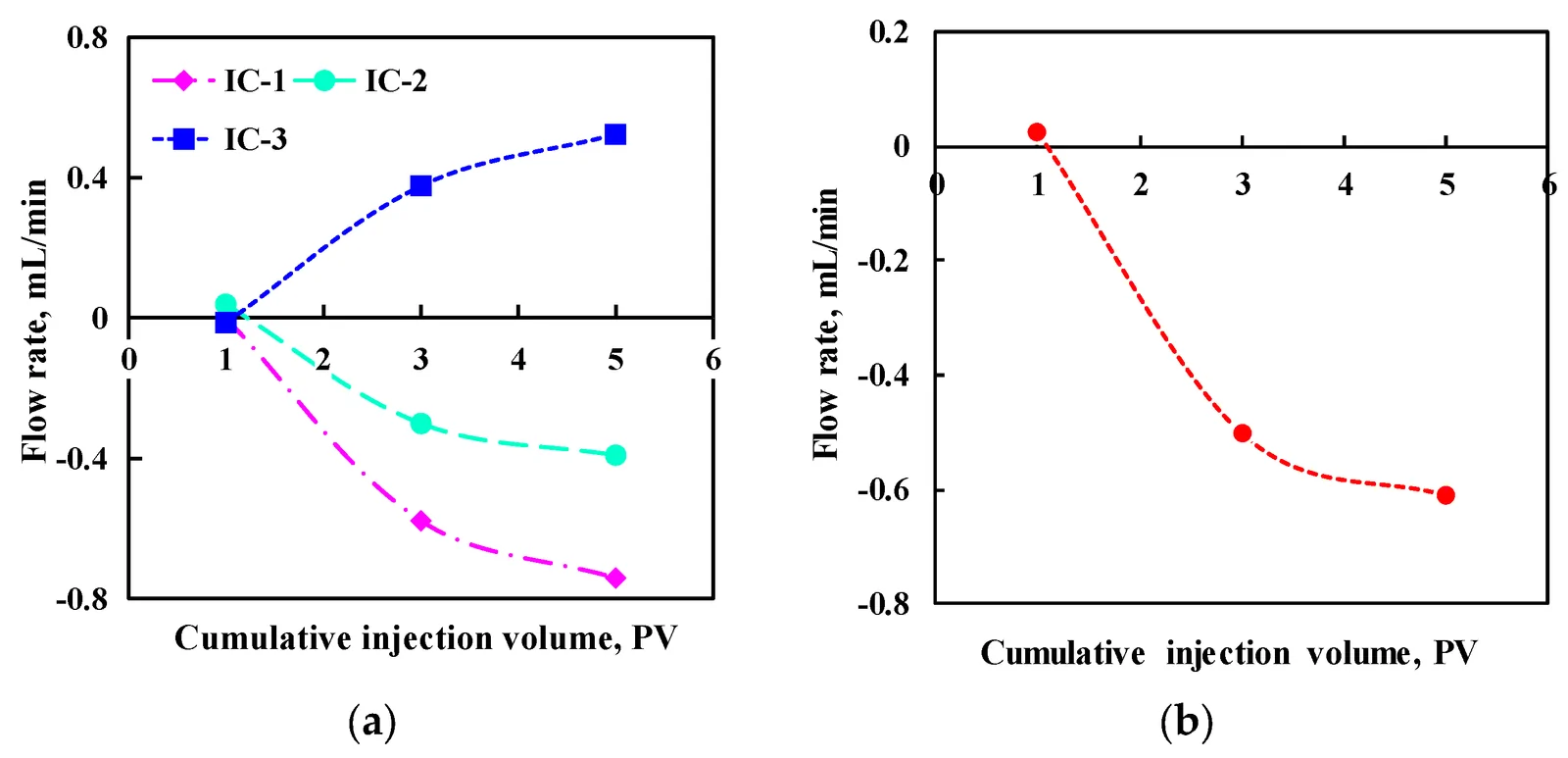
Variation of each interlayer channel flow during DGP injection under different injection volume. (**a**) Flow rate variation of interlayer channels. (**b**) Variation in total interlayer flow rate.

The study of flow rate variations in interlayer channels, influenced by the factors discussed previously, reveals that interlayer pressure difference is a key determinant of interlayer flow. This makes the injection volume of DGPs a critical factor influencing their plugging strength in deep layers. Figure 3 illustrates the changes in interlayer channel flow rates, calculated based on the experimental results from Groups 2, 6, and 7 in Table 1. The analysis shows that as the cumulative injection volume increases from 1 PV to 3 PV, the absolute interlayer flow rate rises from 0.99 mL/min to 4.99 mL/min. However, the growth rate of the interlayer flow rate gradually diminishes, and further increases in injection volume do not reverse the flow direction in interlayer channel-3. Instead, the pressure difference increases, resulting in higher flow rates in this channel. This suggests that increasing the injection volume can enhance the sweep efficiency of injected water to some extent. However, the associated costs and limitations of injection equipment with high volumes must also be considered for field applications.

### 2.2. Interlayer Flow Dynamics During Subsequent Water Injection

One of the primary objectives of profile control is to block the channeling paths, enabling subsequent water to reach the potential areas and mobilize remaining oil. Therefore, this section investigates the flow behavior of injected water between layers following the plugging of channels with DGPs in interlayer pan-connected reservoirs, based on the experimental results.

#### 2.2.1. Influence of Diameter Ratio of DGP to Pore Throat on Interlayer Flow Dynamics During Subsequent Water Injection

**Figure 4:**
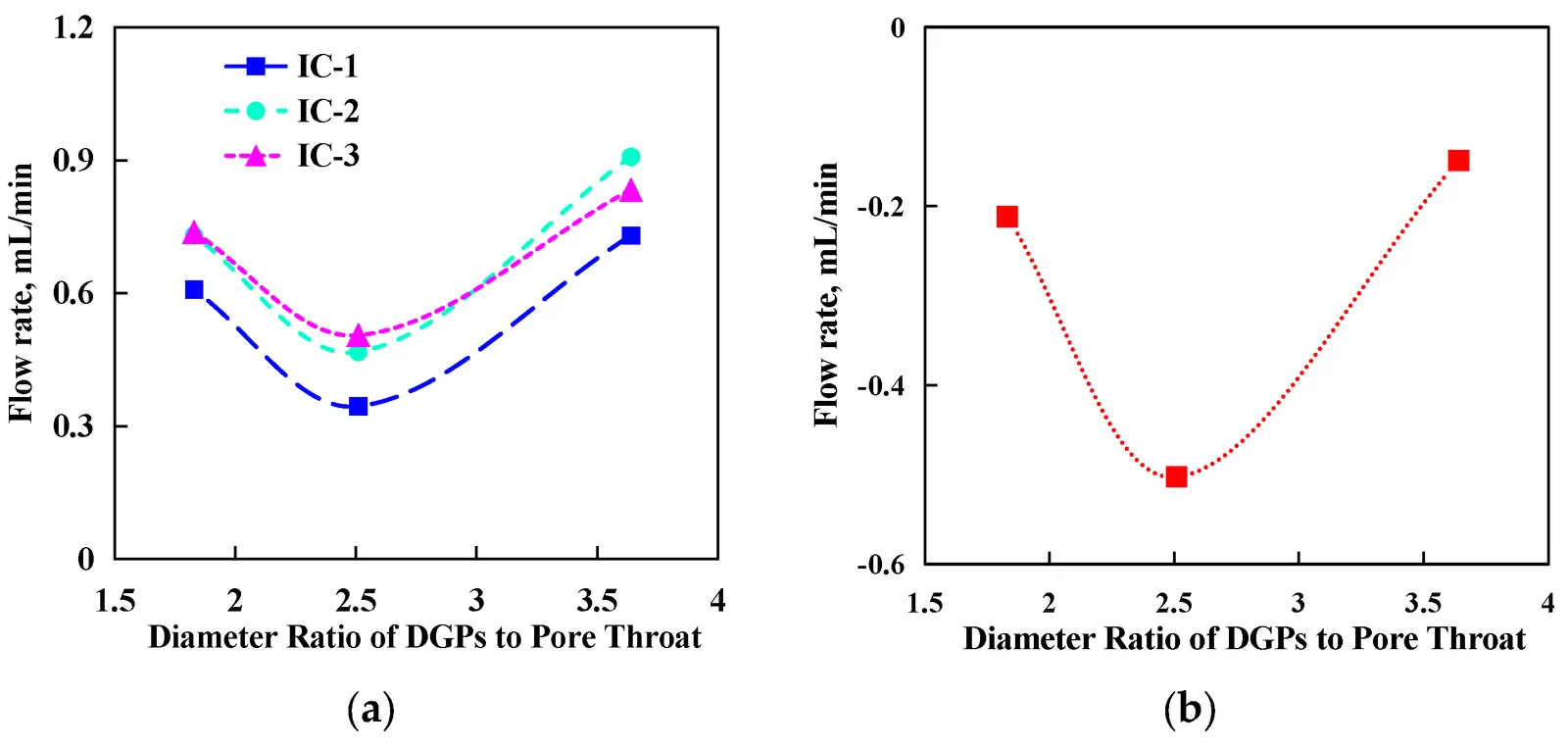
Relationship between interlayer channel flow rate and diameter ratio of DGP to pore throat during subsequent water injection. (**a**) Flow rate variation of interlayer channels. (**b**) Variation in total interlayer flow rate.

A filter cake forms at the entrance of the low-permeability layer after DGP injection [27]. Simultaneously, some particles invade the reservoir due to DGP breakage, resulting in a startup pressure gradient when the low-permeability layer is subsequently injected with water. The median diameter (d50) of the injected DGPs is larger than the median pore throat diameter of the high-permeability layer, which increases the plugging strength at the front end of the high-permeability layer for each diameter ratio. As a result, the subsequent water mainly enters the low-permeability layer. As shown in Figure 4, regardless of the size of the injected DGPs, water flows from the low-permeability layer to the high-permeability layer across all interlayer channels. A larger interlayer flow rate indicates a weaker plugging strength of the particles in the high-permeability layer, allowing subsequent water to enter the high-permeability layer with relatively weak seepage resistance.

It can be concluded that the plugging strength near the injection well in the high-permeability layer is highest for each diameter ratio, resulting in the smallest flow rate in the interlayer channel near the injection well. The plugging effect of particles with a diameter ratio of 2.51 is more uniform within the high-permeability layer, leading to smaller flow rates in all interlayer channels. The total flow rate in this case is 1.32 mL/min, which is less than half of the injected flow rate.

#### 2.2.2. Influence of Injection Rate on Interlayer Flow Dynamics During Subsequent Water Injection

**Figure 5:**
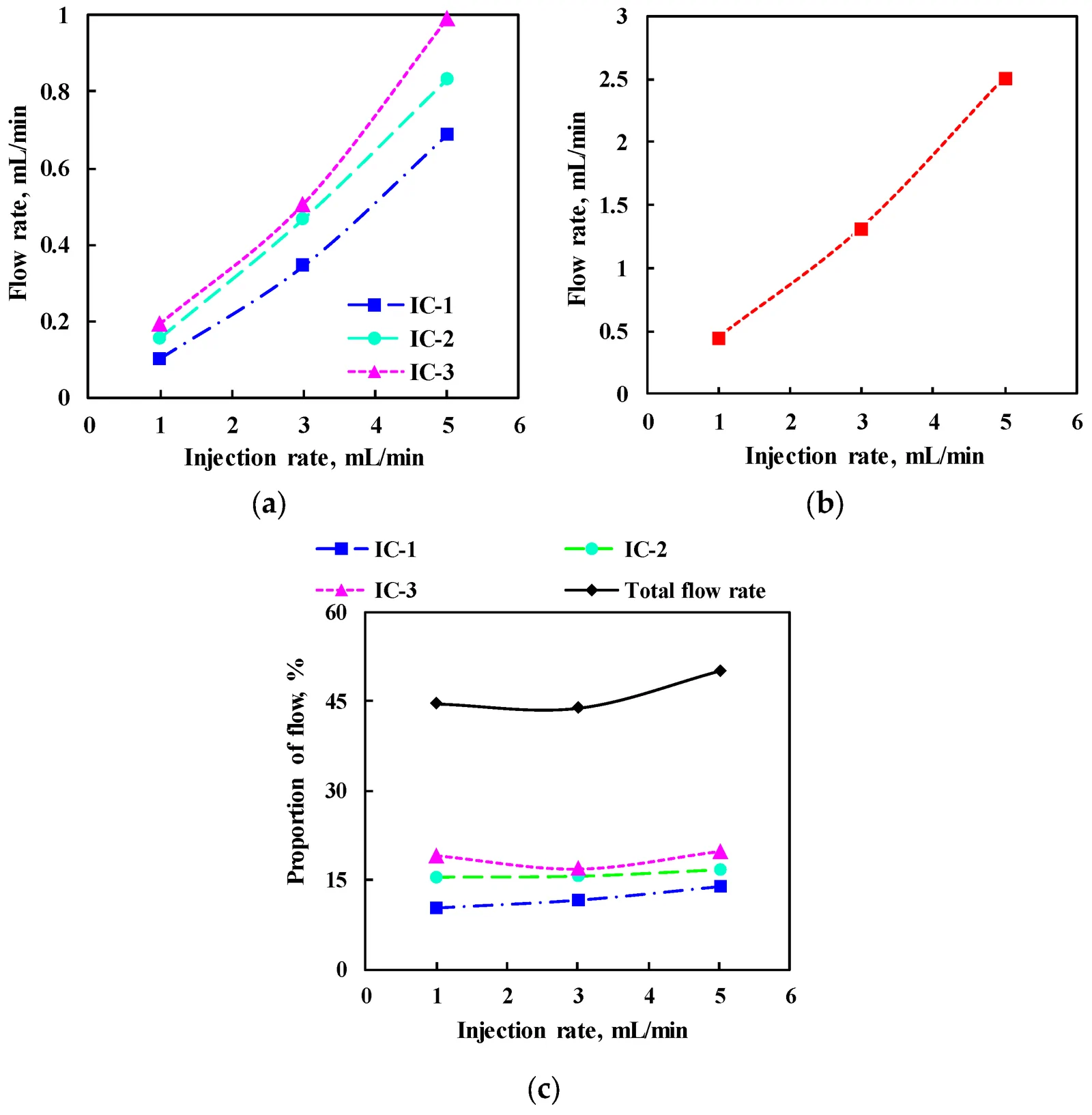
Relationship between interlayer channel flow rate and DGP injection rate during subsequent water injection. (**a**) Flow rate variation of interlayer channels. (**b**) Variation of total interlayer flow rate. (**c**) Proportion of flow rate of each interlayer channel.

The relationship between interlayer channel flow rates and injection rates during subsequent water flooding is shown in Figure 5. Analysis reveals that, similar to the flow directions observed under different particle size-to-pore throat ratios, all interlayer channel flow rates during subsequent water injection are positive, indicating a flow direction from the low-permeability layer to the high-permeability layer. As shown in Figure 5c, the flow rates in the interlayer channels near the injection well (interlayer channel-1) and deeper within the reservoir (interlayer channel-2) increase with increasing injection rates. However, the flow rate in the interlayer channel near the production well (interlayer channel-3) accounts for 16.85% at an injection rate of 3 mL/min, slightly lower than 18.07% at 1 mL/min and 19.78% at 5 mL/min. This variation is due to the fact that at low injection rates, the driving force is insufficient, resulting in weaker deep migration ability, despite less particle crushing and migration. Conversely, at high injection rates, DGPs undergo intense shear and crushing in the front part of the high-permeability layer, weakening the sealing ability of the deep reservoir. Therefore, under both injection rates, the flow rates in the interlayer channel near the production well remain less than 3 mL/min.

#### 2.2.3. Influence of Injection Volume on Interlayer Flow Dynamics During Subsequent Water Injection

**Figure 6:**
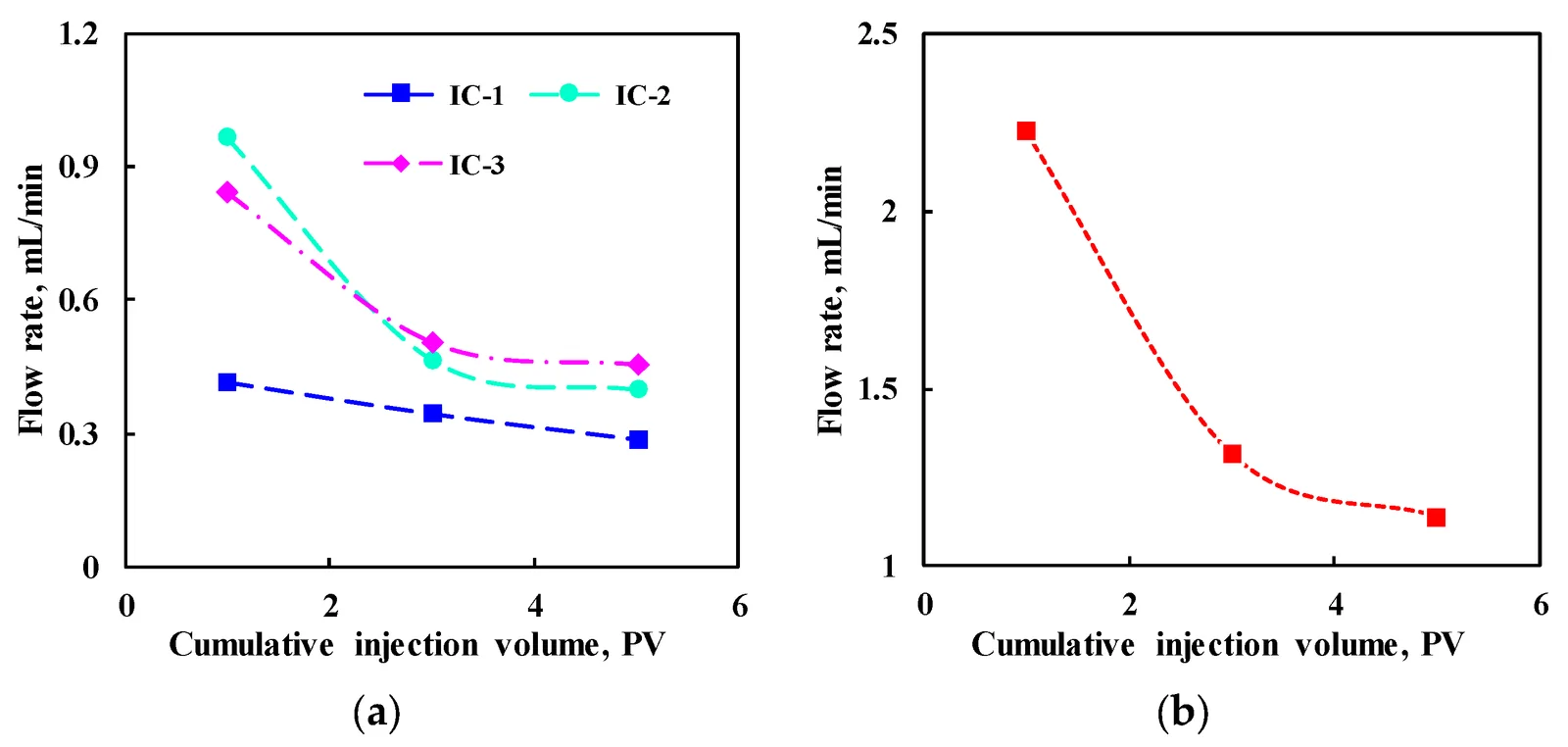
Relationship between flow rate of interlayer channel and DGP injection volume during subsequent water injection. (**a**) Flow rate variation of interlayer channels. (**b**) Variation in total interlayer flow rate.

As shown in Figure 6, with an increasing injection volume, the action intensity of DGPs along the water channeling layer also increases, resulting in more injected water entering the low-permeability layer. Additionally, the seepage resistance in the low-permeability layer is smaller than that in the water channeling layer after the action of DGPs. Specifically, the flow rates of each channel and the total flow rate decrease with increasing injection volume (Figure 6a). After injecting 5 PV of DGPs and conducting subsequent water flooding, the interlayer flow rate can be reduced to 29.6% of the injection rate. Based on the findings from the deep sealing law study, it is observed that the front part of the porous media is highly influenced by the injection volume. Consequently, the flow rate in interlayer channel-1 decreases most rapidly with increasing injection volume. However, when the injection volume is 1 PV, the action intensity in the front part is insufficient to seal interlayer channel-1, resulting in the largest flow rate in this channel. The subsequently injected water mainly bypasses this channel and returns to the water channeling layer. When the cumulative injection volume reaches 3 PV, the seepage resistance in the front part of the water channeling layer becomes sufficiently large, effectively sealing channel-1. As a result, the flow rate in interlayer channel-1 is relatively small, and the injected water bypasses back to the potential layer through interlayer channel-2. Consequently, the flow rate in channel-2 is higher than in the other two channels. Due to the crushing effect, the residual resistance near the production well in the porous media remains small across various injection volumes, leading to the smallest flow rate change in channel-3.

### 2.3. Characteristics of Enhanced Oil Recovery by DGP

#### 2.3.1. Influence of Diameter Ratio of DGP to Pore Throat on Interlayer Flow and Oil Recovery

**Figure 7:**
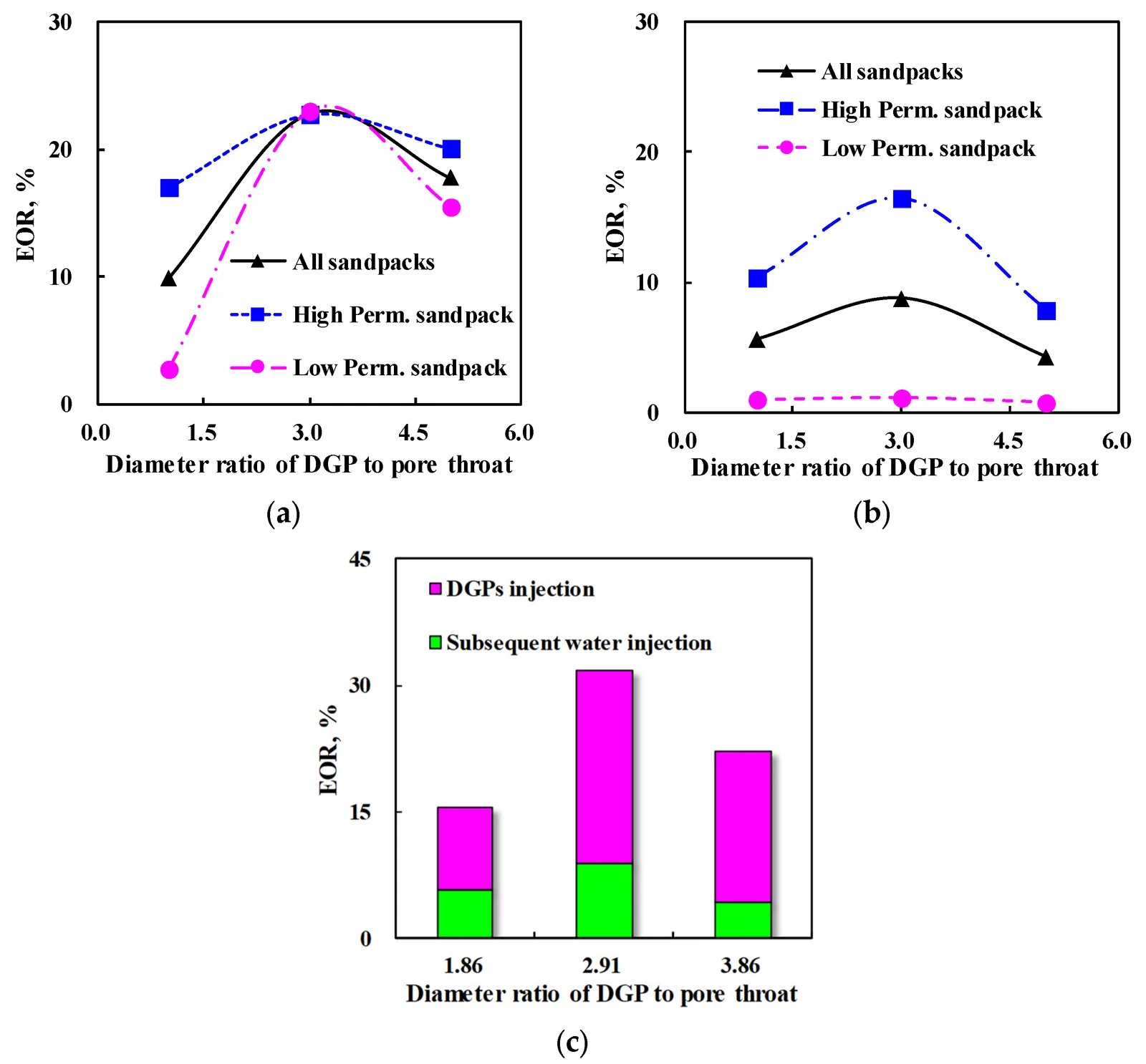
Improvement of recovery degree at different stages under different diameter ratio of DGP to pore throat. (**a**) Stage of DGP injection. (**b**) Stage of subsequent water injection. (**c**) Comparison of enhanced recovery degree in different stages.

As shown in Figure 7a,b, the improvement in oil recovery for each layer during DGP injection and subsequent water flooding is analyzed based on experiments 1 to 3 in Table 2. In both stages, the recovery degree of the high-permeability layer is generally higher than that of the low-permeability layer. However, this does not fully reflect the ability of DGPs to enhance oil recovery in the remaining oil of the high-permeability layer during the injection stage. This discrepancy is primarily due to water carrying oil from the potential layer back to the water channeling layer during the particle injection stage, where it is subsequently produced from the water channeling layer.

As depicted in Figure 7c, the oil recovery improvement during the injection stage of DGPs is significantly higher than during the subsequent water injection stage, regardless of the particle size to pore throat ratio. For instance, when the particle size to pore throat ratio is 2.51, the oil recovery improves by 22.83% during the DGP injection stage, whereas it only improves by 8.83% during the subsequent water flooding stage. In the application and research of DGPs, scholars widely recognize their profile control and oil displacement functions. The enhanced oil recovery characteristics mentioned above are direct manifestations of these functions.

When the diameter ratio is small, the interlayer pressure difference and flow rate during DGP injection are low, resulting in minimal oil recovery improvement during this stage—only 9.85%. In contrast, when the diameter ratio is large, DGPs primarily work in the front part of the high-permeability layer, causing the subsequent water to bypass and return to the high-permeability layer through interlayer channels in the middle of the reservoir. As a result, oil recovery in this stage is the lowest, reaching only 4.35%. When the diameter ratio is 2.51, the final oil recovery in the high-permeability layer reaches 82.19%, which is notably higher than other diameter ratios but still inconsistent with realistic recovery values observed in real reservoirs. This discrepancy arises from interlayer flow, where residual oil from the low-permeability layer is displaced into the high-permeability layer under water flooding and subsequently produced at all stages. In summary, the optimal control effect is achieved when the diameter ratio is 2.51.

#### 2.3.2. Characteristics of Enhanced Oil Recovery Under the Influence of Injection Rate

**Figure 8:**
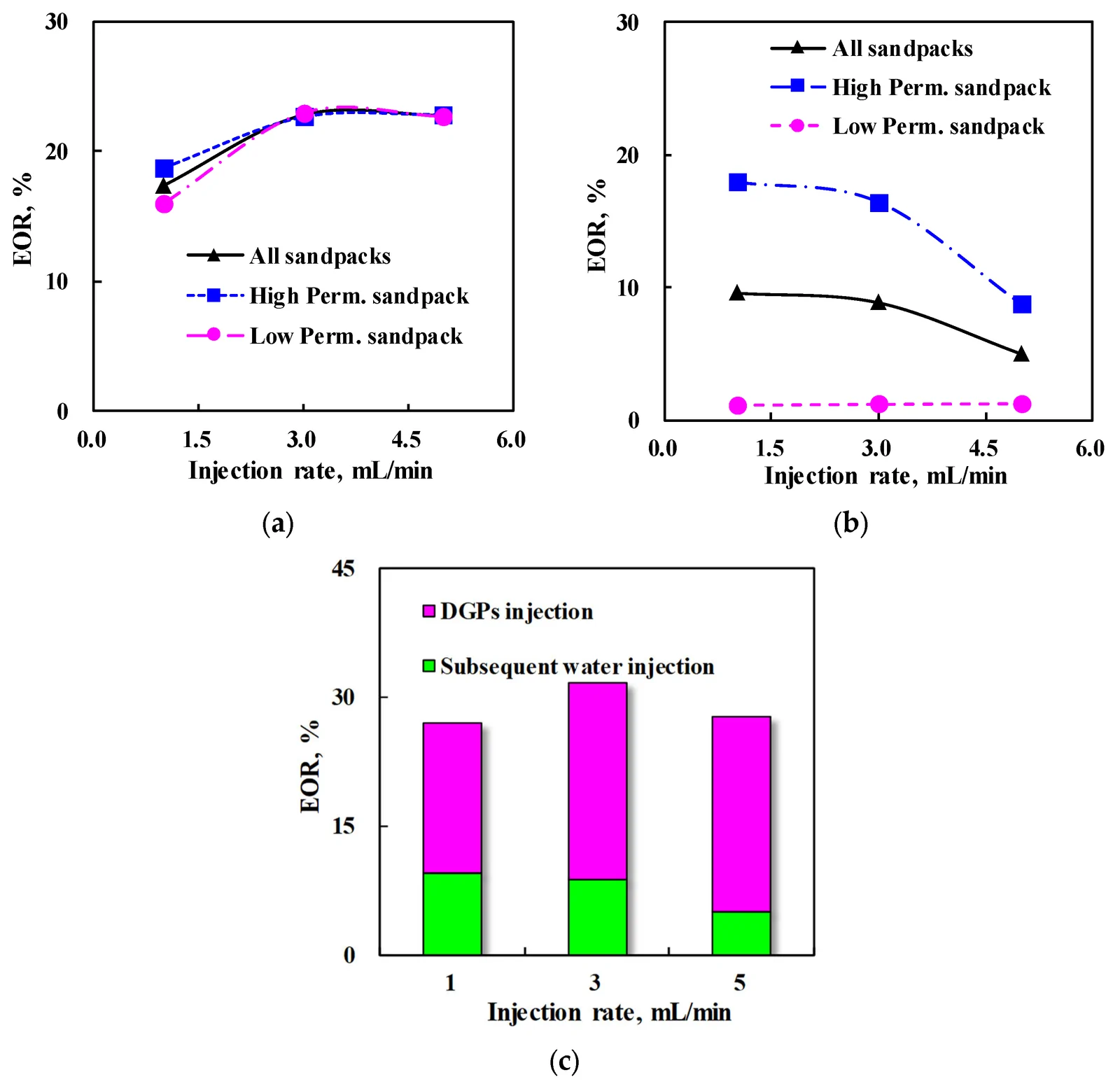
Improvement of recovery degree at different stages under different DGP injection rate. (**a**) Stage of DGP injection. (**b**) Stage of subsequent water injection. (**c**) Comparison of enhanced recovery degree in different stages.

Based on the experiments in Groups 2, 4, and 5 in Table 2, the improvement in oil recovery for each layer during the two stages with varying injection rates is presented in Figure 8. When the injection rate is 3 mL/min, the oil recovery for each layer is highest, with an overall improvement of 22.83% for the entire reservoir. In contrast, under lower injection rates, the overall improvement in oil recovery is only 17.35%.

The improvement in oil recovery during the subsequent water flooding stage is influenced by the blocking strength and penetration depth of particles in the high-permeability layer. Higher injection rates lead to shear breakage in the middle and front parts, while lower injection rates result in insufficient driving force. However, at an injection rate of 1 mL/min, more particles pass through by deformation, leading to higher blocking strength in the high-permeability layer compared to high-rate injection (5 mL/min). Additionally, due to the poor oil displacement efficiency during the injection of deformable gel particles at low injection rates, the residual oil content in the low-permeability layer remains higher. Consequently, the overall oil recovery during the subsequent water flooding stage at a low injection rate reaches 9.57%, whereas it is only 5.01% when the injection rate is 5 mL/min.

#### 2.3.3. Characteristics of Enhanced Oil Recovery Under the Influence of Injection Volume

**Figure 9:**
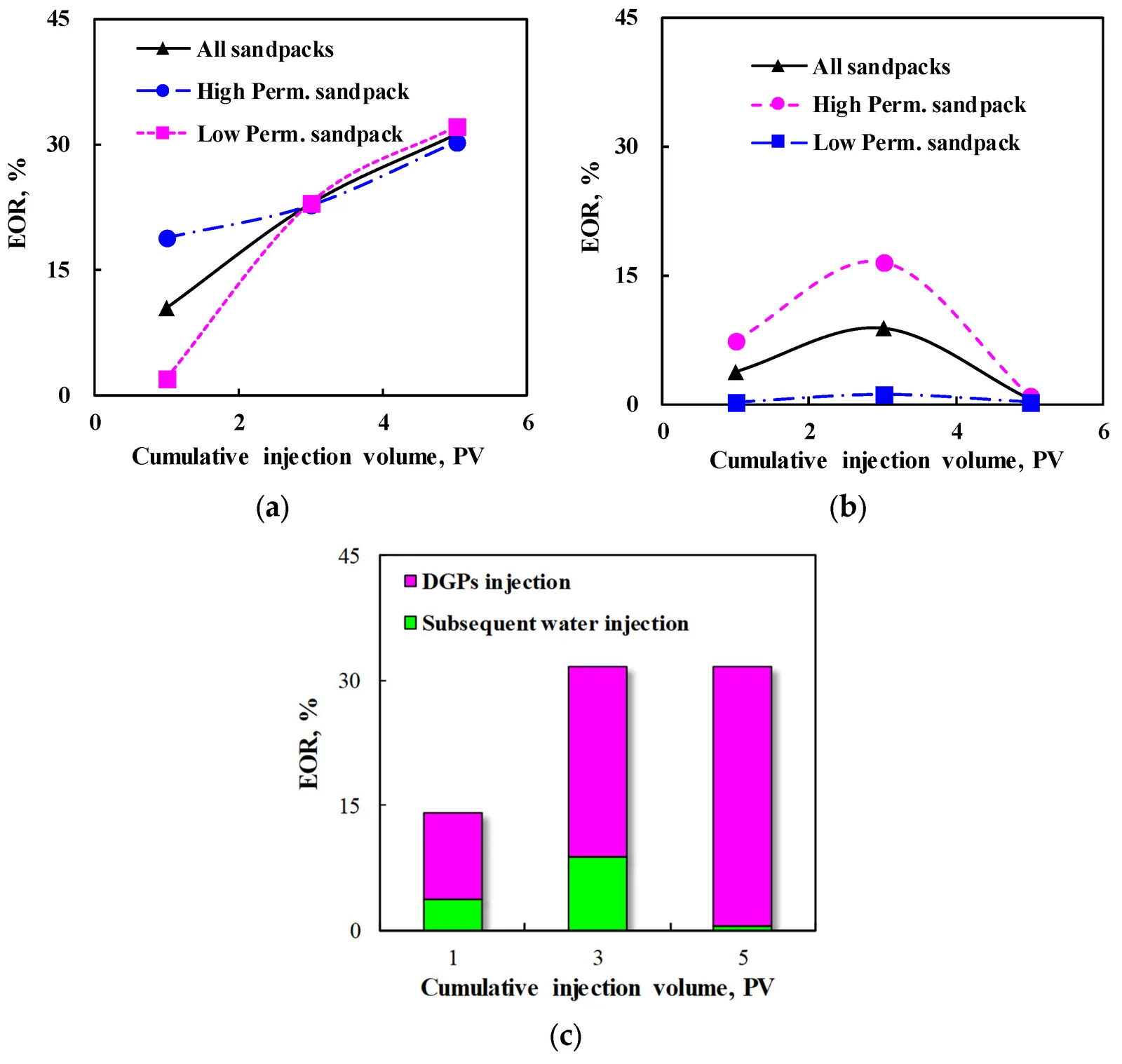
Improvement of recovery degree at different stages under different DGP injection volume. (**a**) Stage of DGP injection. (**b**) Stage of subsequent water injection. (**c**) Comparison of enhanced recovery degree in different stages.

Based on the experimental results of Groups 2, 6, and 7 in Table 2, the characteristics of enhanced oil recovery (EOR) under different injection volumes are presented in Figure 9. The analysis indicates that, across all three injection volumes, the recovery of oil in the high-permeability layer is generally higher than that in the low-permeability layer. This is primarily due to the interlayer channels near the production well consistently facilitating the flow from the low-permeability layer to the high-permeability layer, which results in the residual oil from the low-permeability layer being mobilized into the high-permeability layer and subsequently recovered.

During the injection of DGPs, channels 1 and 2 continuously allowed water from the suspension to enter the potential layers, significantly enhancing oil displacement. However, during subsequent water flooding, injected water consistently bypassed through all three channels, returning to the high-permeability layer. Therefore, when compared to experimental results without interlayer channels [14], the injection of DGPs led to a marked improvement in oil recovery, surpassing the enhancement observed during the subsequent water flooding stage across all injection volumes. The most significant difference was observed when the cumulative injection volume reached 5 PV. This was mainly due to two factors: first, after 5 PV of injection, less residual oil remained during subsequent water flooding, and second, the residual oil was primarily concentrated near the production well in the potential layers. However, due to the relatively weak effect of DGPs in this region and the persistent existence of the final interlayer channel, it was difficult to mobilize the remaining oil from this area.

The enhanced recovery degree of each layer after the injection of DGPs with varying diameter ratio and subsequent water flooding is shown in Figure 7. In both stages, the recovery degree of the high-permeability layer is generally higher than that of the low-permeability layer at each diameter ratio. However, this does not fully reflect the ability of DGPs to improve the recovery of residual oil in the high-permeability layer during the DGP injection stage. A significant factor contributing to this is the flow direction of DGPs during injection. As illustrated in Figure 9, the interlayer pressure difference during DGP injection drives the flow from the high-permeability layer to the low-permeability layer, with water transporting residual oil from the low-permeability layer into the high-permeability layer.

**Figure 10:**
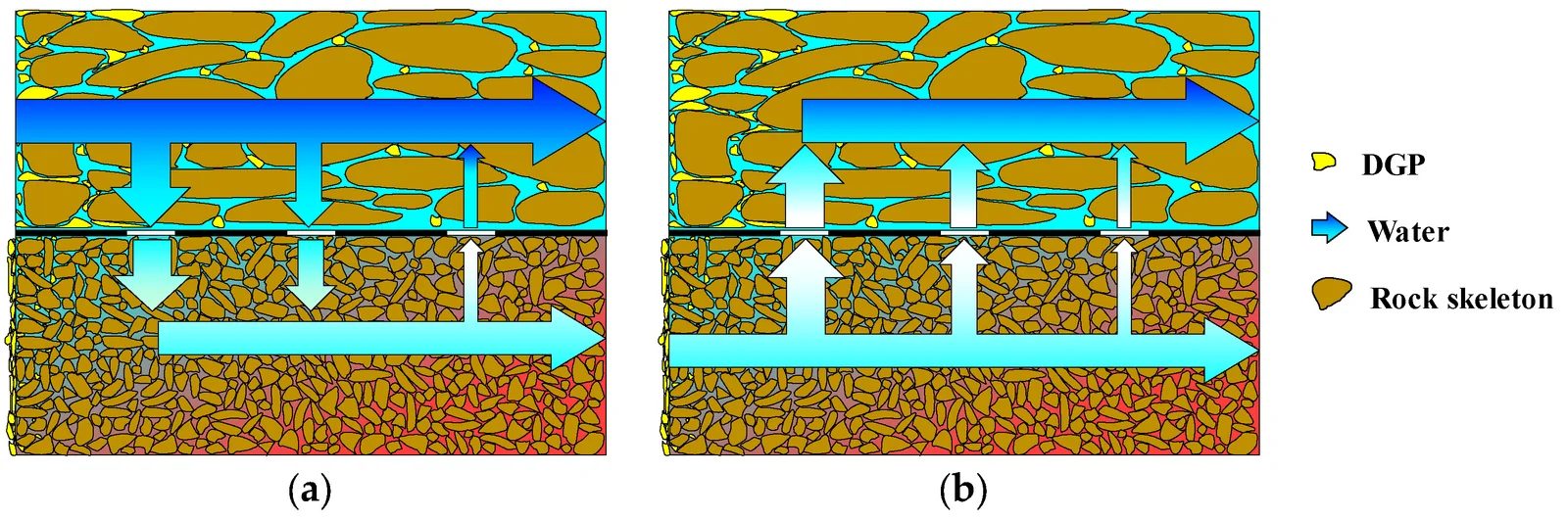
Comparison of interlayer channel flow direction. (**a**) Interlayer channel flow direction during DGP injection. (**b**) Interlayer channel flow direction during subsequent water flooding.

From the results of the three experiments, it is evident that DGPs can effectively control interlayer pan-connected reservoirs in the high-water-cut stage. As shown in Figure 10a, interlayer channel-1 and interlayer channel-2 always flow from the high-permeability layer to the low-permeability layer, with the fluid then returning to the high-permeability layer through IC-3 during the DGP injection stage. In Figure 10b, the flow direction of all interlayer channels during the subsequent water flooding stage is from the low-permeability layer to the high-permeability layer.

Based on the recovery improvement characteristics, the control mechanism of DGPs in interlayer pan-connected reservoirs during the high-water-cut stage can be summarized as follows: during the DGP injection stage, the pressure in the water channeling layer is significantly higher than in the remaining oil enrichment layer due to deep migration. This causes the water in the particle system to enter the potential layers, with most of the fluid then flowing to the water channeling layer and being recovered from this layer after mobilizing the remaining oil from the potential layer.

## 3. Conclusions

This study develops a physical model of an interlayer pan-connected reservoir, quantitatively characterizes interlayer flow, and explores the regulatory effects of deformable gel particles (DGPs) under various influencing factors. The main conclusions are as follows:

During DGP injection, the water phase in the DGP suspension passes through two interlayer channels in the middle and front parts, entering the potential layer from the water breakthrough layer and bypassing the channel near the production well to re-enter the water breakthrough layer. Experimental results indicate that interlayer flow rates are relatively high when injection volumes are large, and particle sizes and injection rates are moderate. However, the interlayer channel near the production well consistently flows from low permeability to high permeability. Under various injection conditions, the average increase in oil recovery during this stage can reach 19.74%.

During subsequent water flooding, all interlayer channels flow from the potential layer, enriched with residual oil, to the water breakthrough layer. Low injection rates limit deep migration of DGPs, while high rates intensify particle breakage. Additionally, smaller particles have weaker blocking capabilities, while larger particles primarily act in the middle and front parts of the porous media. These factors collectively result in a higher interlayer flow rate during this stage, which significantly impairs oil displacement. At this stage, the average increase in oil recovery under various injection conditions is 6.17%.

DGPs can effectively regulate widely interconnected reservoirs with interlayer barriers. Their regulatory mechanism is primarily reflected in the following: during injection, the migration of particles in the deep sections of the water breakthrough layer leads to an overall pressure increase in this layer, which, in turn, propagates the water phase to the potential layer and mobilizes the residual oil within it. Under the experimental conditions of this study, the optimal regulatory effect is achieved when DGPs with a particle size-to-pore throat ratio of 2.51 are injected at a rate of 3 mL/min for 3 pore volumes.

Despite their deep migration capability, DGPs’ relatively large particle size limits their application in low-permeability reservoirs. Future studies will focus on deep profile control and displacement in interlayer channels of low-permeability reservoirs, as well as investigating reservoir damage caused by the profile control agents, aiming to optimize DGP properties for broader reservoir adaptability. A key limitation is the lack of simulation for specific reservoir environments of a particular oilfield. Future research will use real cores from a specific oilfield and conduct experiments under temperature and salinity conditions matching the reservoir to enhance field relevance.

## 4. Materials and Methods

### 4.1. Materials

**Figure 11:**
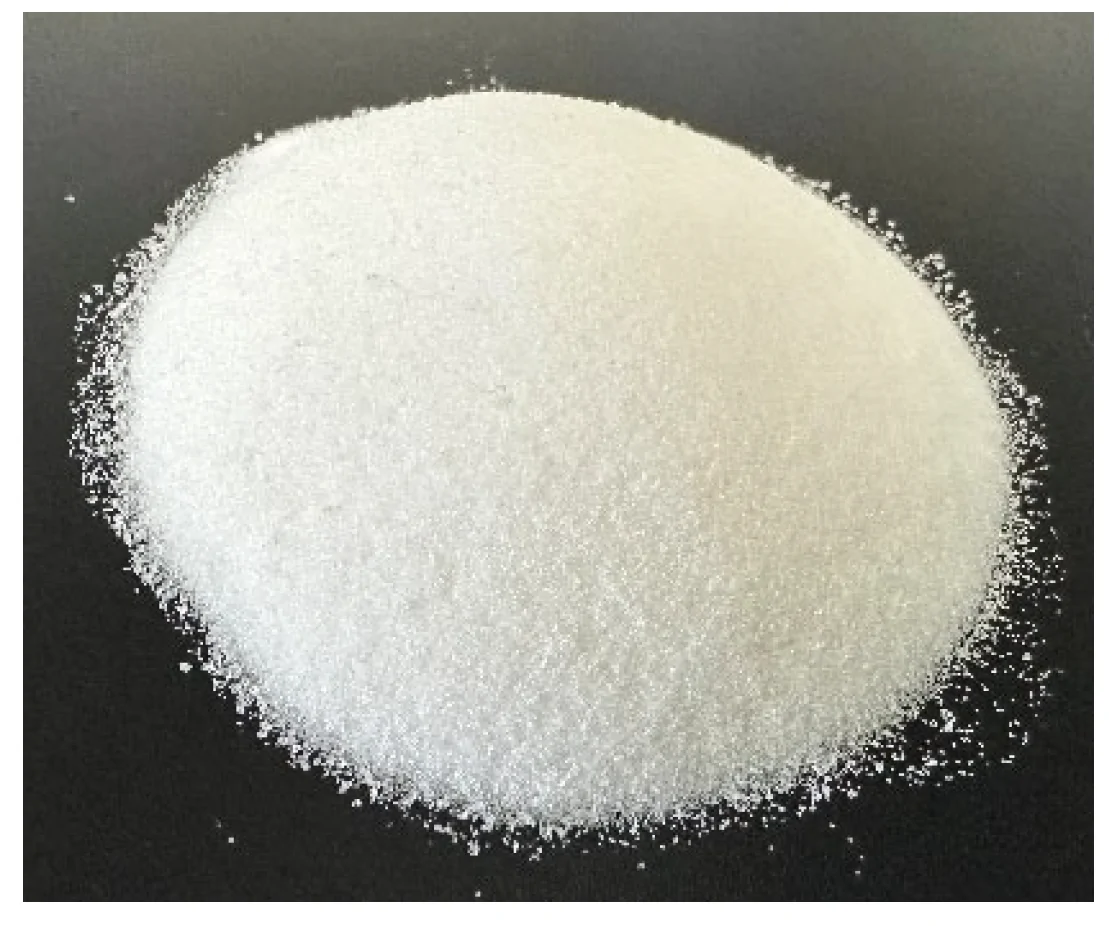
Deformable gel particles (DGPs).

Deformable gel particles (DGPs) are primarily composed of acrylamide, polyacrylamide, acrylonitrile monomer, polyvinyl alcohol, N-methylenebisacrylamide, and ammonium persulfate-sodium bisulfite, and were produced by Guangzhou Bofeng Chemical Science and Technology Co., Ltd. (Guangzhou, China). The DGPs are dry powder with a particle size range of 140–170 mesh before water absorption and expansion as shown in Figure 11.

**Figure 12:**
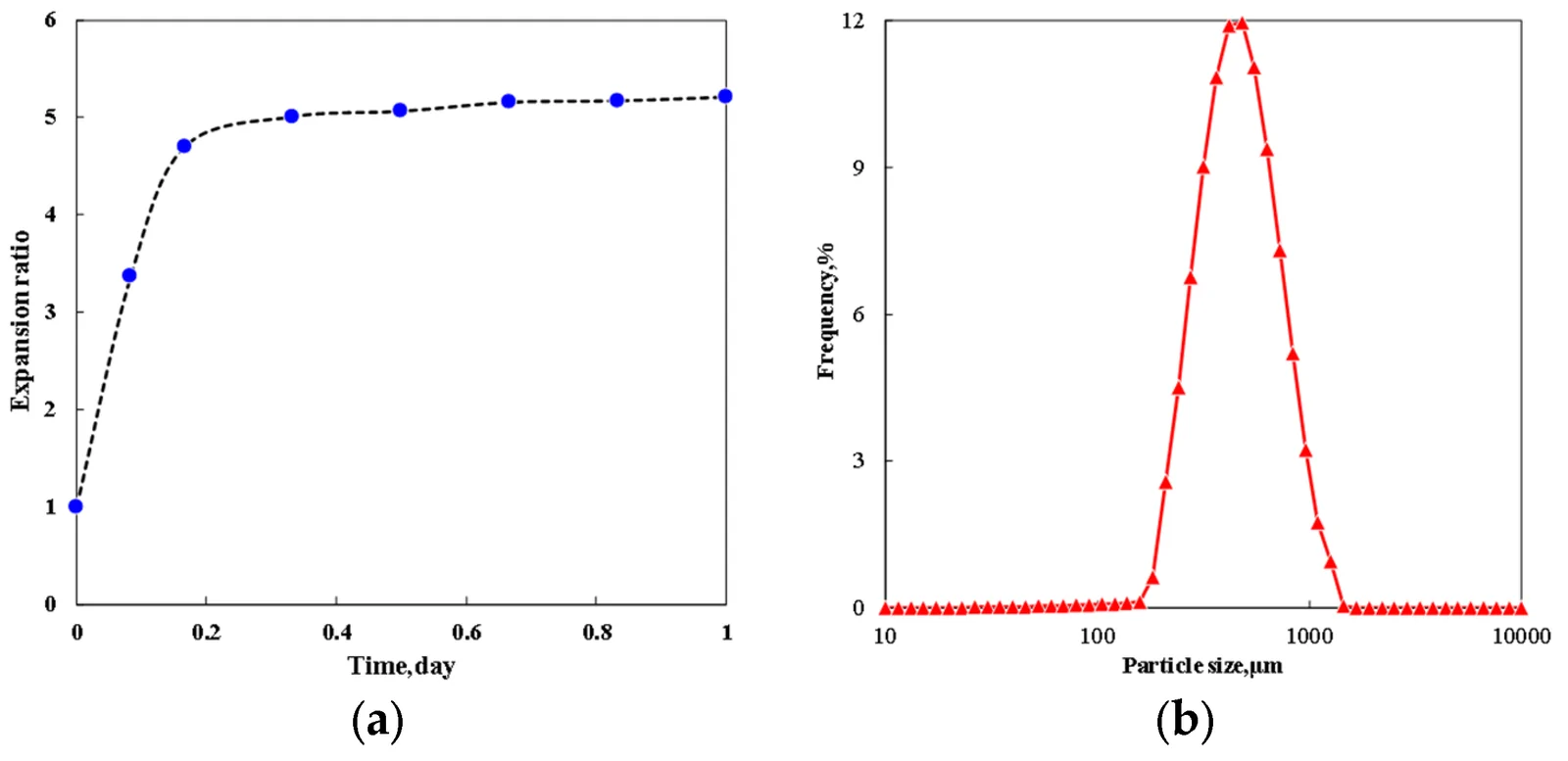
Particle size distribution of DGPs. (a) The swollen features of DGPs, (**b**) particle size distribution after swelling.

The change in particle size of DGPs after water absorption and expansion is shown in Figure 12a. The particle size distribution after full expansion is shown in Figure 12b. DGPs expanded 5.06 times after 12 h of water absorption, with no further size change thereafter. After that, the particle size does not change. And the particle size was measured via dynamic light scattering (Malvern Mastersizer 3000, Malvern, UK). Table 3 shows the typical properties of the DGP sample at 2000 mg/L.

Because the influence of salinity is not considered in this study, distilled water was used in the experiment. For the enhanced oil recovery (EOR) experiments, 25#-vash oil (877.6 kg/m^3^,10.5 mPa.s) was used.

### 4.2. Physical Model

A sandpack was used to construct a physical model of porous media for the experiments. The sandpack has a length of 60 cm and a diameter of 3.8 cm. Three interfaces are located on one side of the sandpack for pressure monitoring. Pressure sensors with a measurement range of 0–5 MPa are installed at the entrance and along the three interfaces. Filters are installed at both the outlet and interfaces to prevent quartz sand from flowing out, ensuring the accuracy and uninterrupted operation of the experiment.

**Figure 13:**
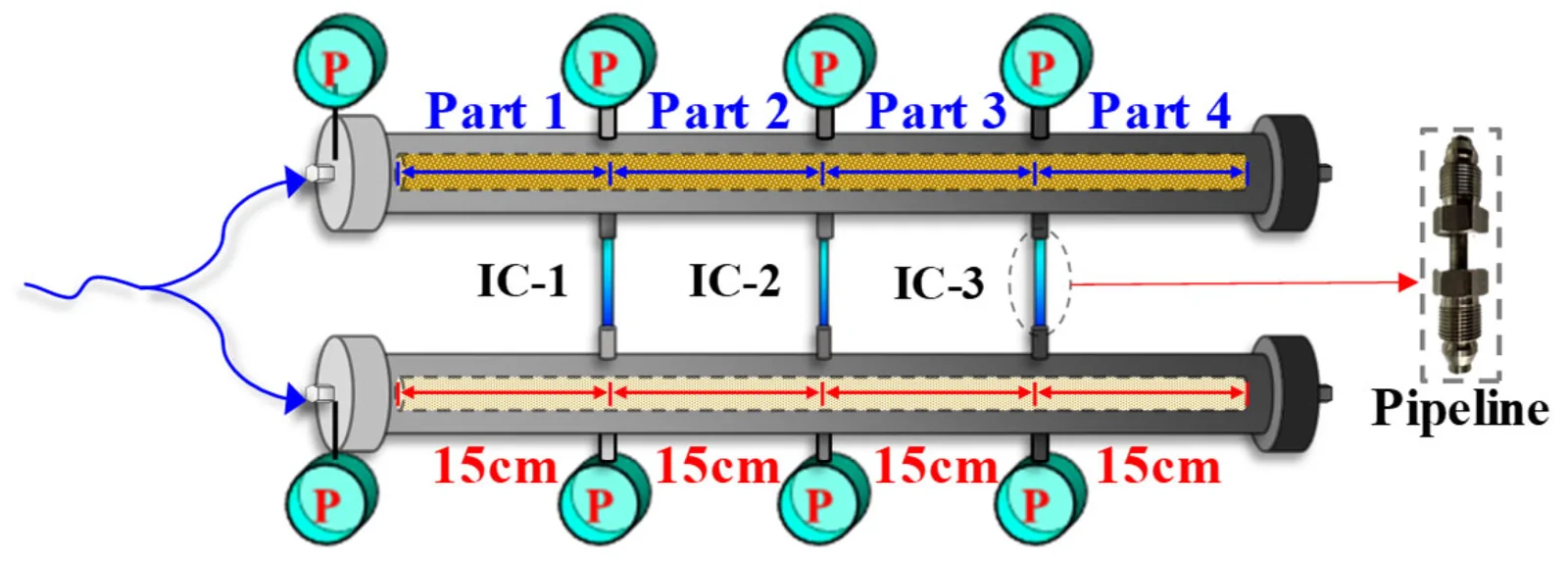
Physical model considering interlayer channels.

The sandpack dimensions are consistent with those used in the DGP flow experiments. As shown in Figure 13, each sandpack has three interfaces, which are used for pressure monitoring and interlayer channel simulation. The sandpacks are connected by a thicker pipeline with an inner diameter of 6 mm, simulating the interlayer channels. These interfaces represent the flow channels near the injection well, the reservoir depth, and the production well. A filter screen is installed at the outlet, and pressure sensors are positioned at the entrance of the high-permeability sandpack, all interfaces, and the entrance of the low-permeability sandpack. For the low-permeability sandpack, pressure sensors (0.01% F.S.) with a measurement range of 0–0.110 MPa are used along the three interfaces.

### 4.3. Experimental Procedures and Steps

**Figure 14:**
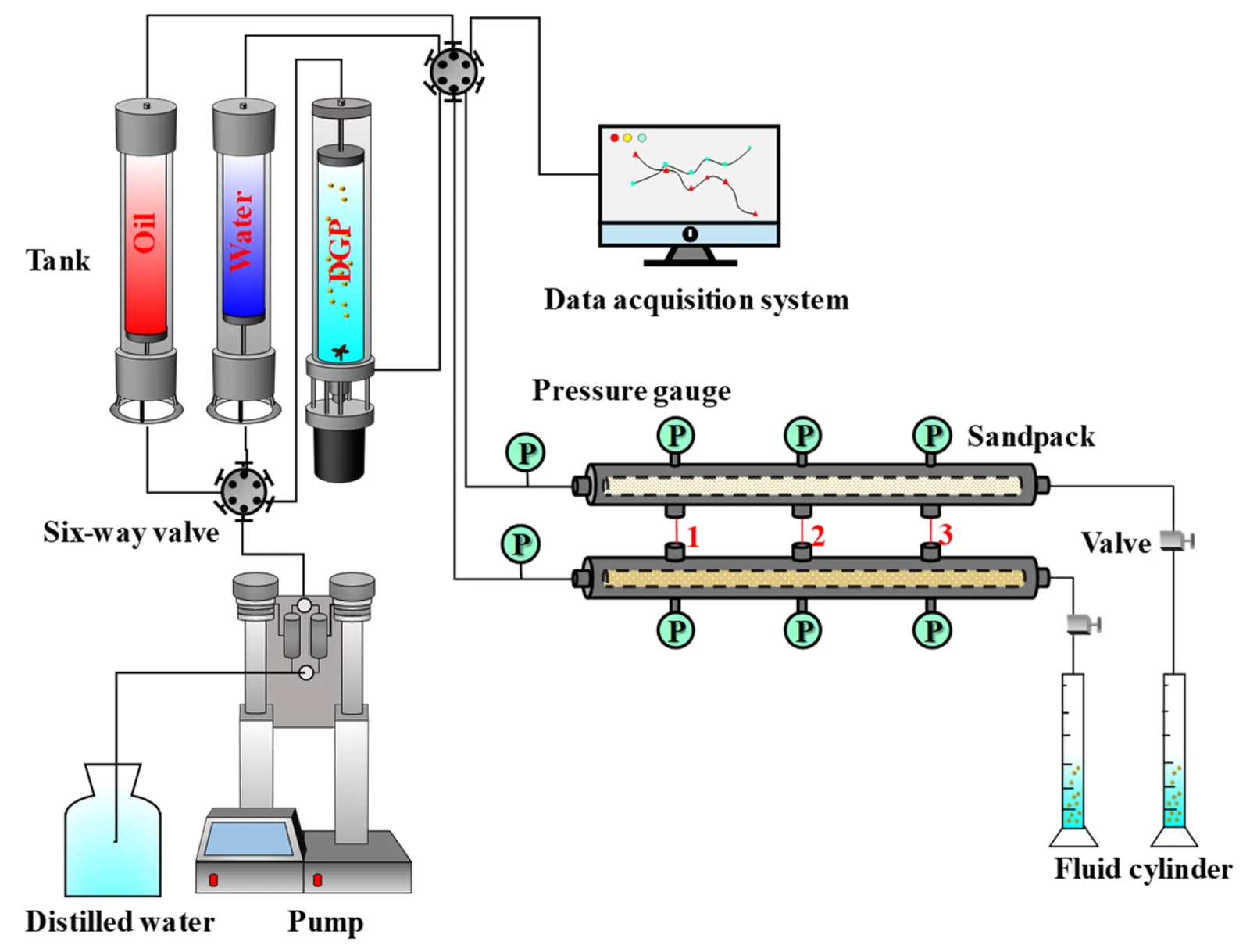
Physical simulation process of DGP flow experiments.

The high- and low-permeability sandpacks were filled with quartz sand (1–10 mesh and 100–120 mesh, respectively), and the experimental setup was arranged as shown in Figure 14. To accurately characterize the particle regulation behavior in an interlayer-connected reservoir, both flow and EOR experiments were conducted simultaneously using nearly identical parameters, as outlined in Table 1 and Table 2. In Table 3 and Table 1: PV refers to the ratio of the injected DGP suspension to the pore volume. And the diameter ratio of DGP to pore is the ratio of the median particle size of DGPs to the pore throat diameter.

The main experimental steps are as follows:

◼ Equipment verification and connection: The air-tightness and connectivity of the experimental setup were thoroughly checked, focusing on the flow channel between the two sandpacks. The device was assembled as shown in Figure 14. To prevent DGP sedimentation, a magnetic stirring device was incorporated into the DGP tank.
◼ Physical model creation: Each sandpack was filled with the appropriate mesh-size quartz sand, as specified in Table 1, Table 2 and Table 3. The sand was pre-screened and thoroughly cleaned to ensure that smaller particles did not interfere with the experiment.
◼ Water saturation: The model was saturated at an injection rate of 1 mL/min. The porosity of the sandpack was determined based on the volume of injected water, and permeability was measured at an injection rate of 3 mL/min.
◼ Oil Saturation (EOR experiments only): Oil saturation was performed at the same rate as water saturation, and ceased once the oil-water recovery rate at the outlet stabilized post-water saturation. The oil and water saturations of the sandpacks were calculated based on the volume of the injected oil.
◼ Water flooding (EOR experiments only): Water injection continued until the water cut in one of the sandpacks reached 96%. The volume of oil and water produced from both sandpacks was recorded every 10 min, and pressure changes during water flooding were monitored.
◼ DGP injection into the model: A DGP suspension (2000 mg/L) was prepared and allowed to absorb water and fully expand for 12 h. Subsequently, the suspension was injected into the sandpacks at a rate of 3 mL/min, with varying pore volumes (PV).

## 5. Calculation Method of Interlayer Channel Flow Rate

Bai [16], Elsharafi [20], and Imqam [28] proposed that when the diameter ratio of DGP to pore throat exceeds 17, or when the formation permeability falls below 100 mD, a filter cake forms at the entrance of the sandpack. Even if the DGP infiltrates into the formation, the penetration distance remains limited. Consequently, only the final three parts of the low-permeability sandpack experience water flow in the injection direction.

**Figure 15:**
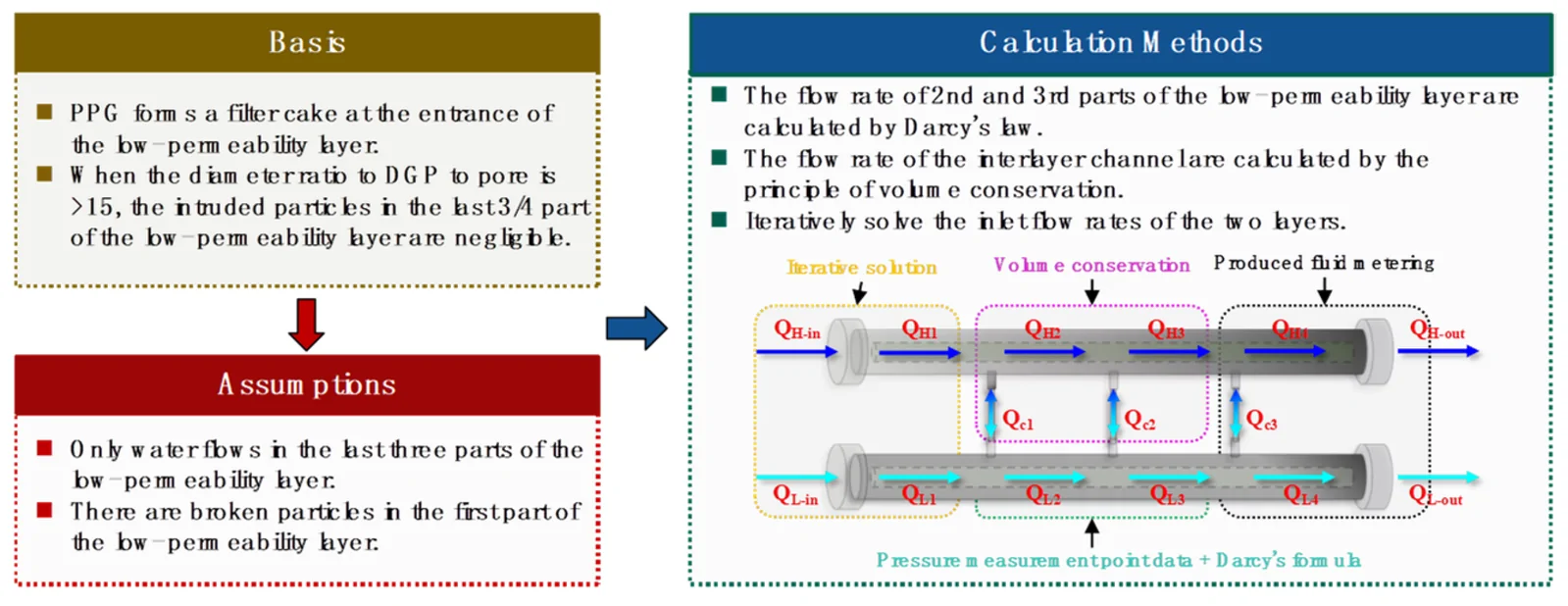
Schematic diagram of interlayer channel flow rate calculation method.

Experiments can obtain the pressure at each measuring point in the low-permeability sandpack. The flow rates of the two middle parts of the low-permeability tube at each moment (labeled as *Q*_L2_ and *Q*_L3_ in Figure 15) can be calculated. Taking *Q*_l2_ as an example, substitute the pressure difference (∆*P*_l2_) measured by pressure measuring points *P*_6_ and *P*_7_ into Darcy’s formula. The calculated *Q*_L2_ is as follows:

(1) $$Q_{L2}=\frac{kA\Delta P_{L2}}{\mu L}=\frac{kA(P_{6}-P_{7})}{\mu L}$$

*L* is the sandpack length (cm), *μ* is the dynamic viscosity of water (cp), *A* is the cross—sectional area (cm^2^), and *k* is permeability (μm^2^).

**Figure 16:**
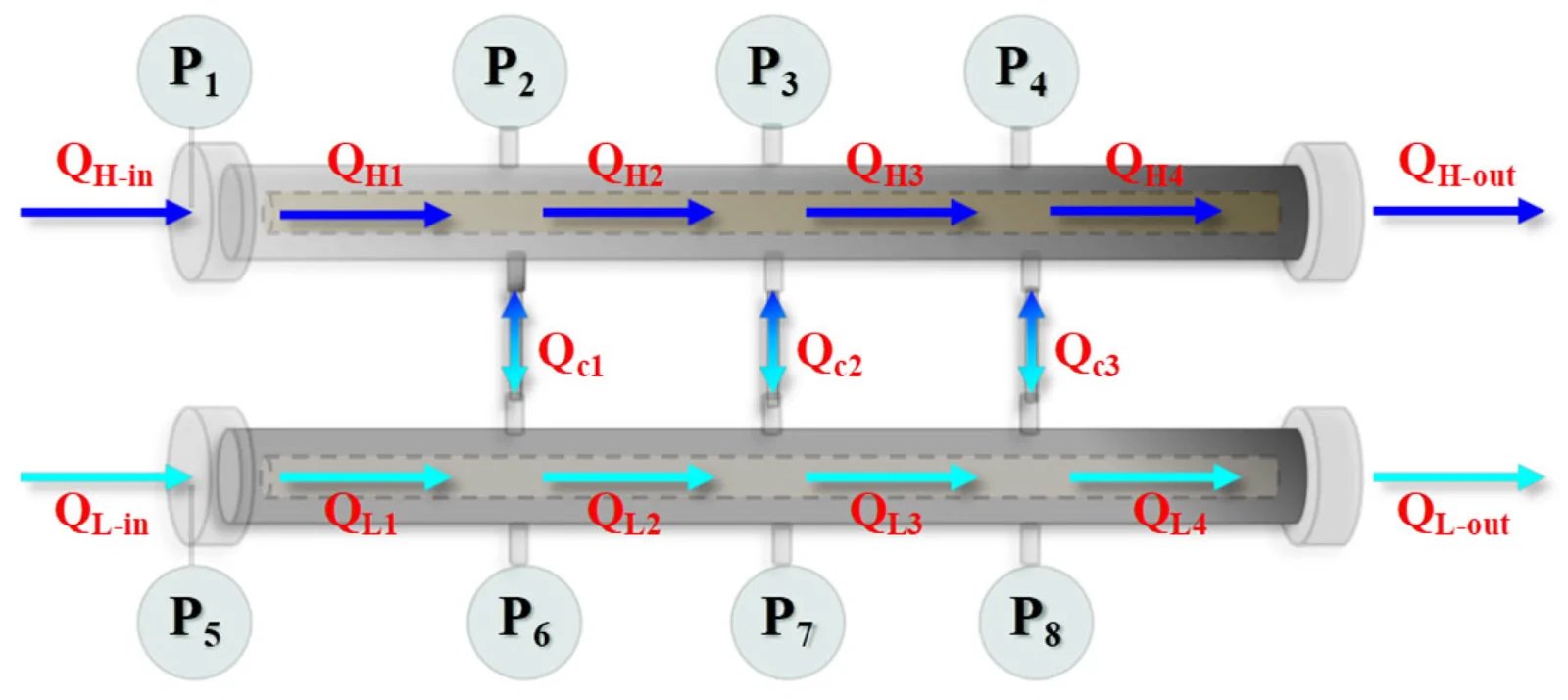
Schematic diagram of flow calculation for interlayer channels.

The pressure difference and permeability can be incorporated into Darcy’s Law to estimate the flow rate for the central two parts of the low-permeability sandpack. The outlet flow rates for the last part of both sandpacks (part 4 in Figure 16), denoted *Q*_Hout_ and *Q*_Lout_, can be determined experimentally by measuring the produced liquid. However, the filter cake affects the flow rate at the entrance of the low-permeability layer, preventing the calculation of *Q*_Lin_ based on the pressure at the entrance of the low-permeability sandpack. Assuming the flow direction from the low-permeability sandpack to the high-permeability sandpack is positive, and applying the principle of volume conservation, the flow rates for the last two flow channels can be expressed as follows:

(2) $$Q_{C3}=Q_{L2}-Q_{\mathrm{Lout}}$$

(3) $$Q_{c2}=Q_{L1}-Q_{L2}$$

Furthermore, the flow rates *Q*_h1_ and *Q*_h2_ for the middle two parts of the high-permeability sandpack can be determined as follows

(4) $$Q_{H2}=Q_{\mathrm{Hout}}+Q_{c3}$$

(5) $$Q_{H1}=Q_{H2}+Q_{H2}$$

Finally, *Q*_Lin_, *Q*_Hin_, and *Q*_c1_ can be iteratively solved using the following relations:

(6) $$Q_{\mathrm{Hin}}=Q_{c1}+Q_{H1}$$

(7) $$Q_{\mathrm{Hin}}=Q_{c1}+Q_{H1}$$

(8) $$Q_{\mathrm{Lin}}+Q_{\mathrm{Hin}}=Q_{\mathrm{Lout}}+Q_{\mathrm{Hout}}=3\mathrm{mL}/\min$$

## Figures and Tables

**Table 1:** Plugging experimental parameters of interlayer pan-connected reservoir.

No.	Permeability, μm^2^	Porosity	Injection Rate, mL/min	Diameter of Pore, μm	Diameter Ratio of DGP to Pore	Cumulative Injection Volume, PV
1	32.01	0.35	3	162.30	1.83	3
	0.87	0.28		29.91	9.93	
2	30.69	0.30		171.65	2.51	
	0.79	0.27		29.03	16.99	
3	30.91	0.31		169.46	3.64	
	0.82	0.28		29.04	21.24	
4	30.97	0.30	1	172.43	2.43	3
	0.79	0.29		28.01	17.39	
5	32.1	0.34	5	164.90	2.58	
	0.89	0.27		30.81	15.93	
6	31.08	0.32	3	167.25	2.52	1
	0.78	0.28		28.32	17.22	
7	29.93	0.30		169.51	2.43	5
	0.72	0.29		26.74	17.92	

**Table 2:** Experimental parameters for improving recovery degree by DGPs in interlayer pan-connected reservoir.

No.	Permeability, μm^2^	Porosity	Injection Rate, mL/min	Diameter of Pore, μm	Diameter Ratio of DGP to Pore	Initial Oil Saturation	Cumulative Injection Volume, PV
1	31.43	0.34	3	163.17	1.86	0.69	3
	0.85	0.27		30.11	10.09	0.61	
2	30.88	0.33		164.16	2.60	0.70	
	0.79	0.29		28.01	17.83	0.69	
3	29.98	0.33		161.75	3.86	0.72	
	0.83	0.26		30.32	20.58	0.60	
4	30.07	0.31	1	167.14	2.78	0.69	3
	0.77	0.27		28.66	10.36	0.58	
5	31.85	0.32	5	168.93	2.63	0.72	
	0.82	0.29		28.54	21.62	0.60	
6	28.96	0.31	3	164.03	2.57	0.71	1
	0.78	0.27		28.84	16.91	0.60	
7	31.74	0.31		171.72	2.39	0.63	5
	0.80	0.29		28.19	17.01	0.74	

**Table 3:** Typical properties of DGPs.

Properties	Values
Moisture Content (%)	<5
Apparent Bulk Density (g/mL)	0.90–0.95
Viscosity (mPa.s)	45.14
pH Value	6–8
Fully swelling Time (h)	12

## Data Availability

The original contributions presented in this study are included in the article. Further inquiries can be directed to the corresponding author.
